# Complete genome sequence of *Lentilactobacillus buchneri* subsp. *silagei* MGR2-32 isolated from guinea grass silage in Okinawa, Japan

**DOI:** 10.1128/mra.00695-23

**Published:** 2024-02-28

**Authors:** Rei Irimajiri, Meimi Kuwabara, Shun Togo, Yasuhiro Fujino, Masanori Honsho, Shiro Mawatari, Takehiko Fujino, Katsumi Doi

**Affiliations:** 1 Microbial Genetic Division, Institute of Genetic Resources, Faculty of Agriculture, Kyushu University, Fukuoka, Japan; 2 Department of Neuroinflammation and Brain Fatigue Science, Graduate School of Medical Sciences, Kyushu University, Fukuoka, Japan; 3 Institute of Rheological Functions of Food, Fukuoka, Japan; SUNY College of Environmental Science and Forestry, USA

**Keywords:** silage, guinea grass, *Lentilactobacillus buchneri*, subtropical environment

## Abstract

The genome sequence of *Lentilactobacillus buchneri* subsp. *silagei* MGR2-32, isolated from guinea grass silage, is 2,540,137 bp, has a GC content of 44%, and contains 2,393 predicted protein-coding genes. Pairwise average nucleotide identity and digital DNA-DNA hybridization values between MGR2-32 and the type strain were 99.75% and 99.90%, respectively.

## ANNOUNCEMENT

*Lentilactobacillus buchneri* subsp. *silage* was recently proposed as a new subspecies of *Lactobacillus buchneri* (1) and was subsequently reclassified (2). This subspecies was isolated from silage, and the type strain was isolated from Japanese rice grain silage. Although *L. buchneri* is an important starter culture in the food industry, it is also a contaminant in beer breweries, living and working in diverse environments (3).

Strain MGR2-32 was isolated from guinea grass ensiled in a bottle silo for 1 year in Okinawa, Japan, alongside other thermotolerant lactic acid bacteria, in an anaerobic jar at 37°C on GYP agar medium (0.2% peptone, 0.1% yeast extract, 0.8% glucose, 0.1% sodium acetate, 0.005% MnCl_2_, 1.8% agar, and 1% CaCO_3_) (4, 5). For growth of the strain, MRS broth (BD Difco) and 2.0% agar plates were used at 37℃. The strain was stored as glycerol stock in a −80°C deep freezer until use. Strain MGR2-32 was purified for genomic analysis according to Marmur’s method with labiase as the lytic enzyme (6).

Sequencing involved the combination of short and long reads. Short-read sequencing libraries were constructed using the NEBNext Ultra DNA Library Prep Kit from Illumina (cat. E7370) and sequenced using a NovaSeq system (Illumina, USA). A total of 10,517,238 reads with 1,577,585,700 bases were generated with an average insert size of 148 bp and a spot length of 300 bp with 2 × 150 bp paired ends. Low-quality bases (*Q* scores < 15) were trimmed, and short reads (<36 bp) were removed using Trimmomatic version 0.38 (http://www.usadellab.org/cms/?page=trimmomatic) (7). Long-read sequence libraries were constructed using a 1D Ligation Sequencing Kit (SQK-LSK109), and sequencing was performed on the PromethION (ONT, UK) system using an R9.4.1 flow cell. No size selection was performed prior to library preparation. A total of 2,633,020 reads with 19,657,745,370 bases were generated using Guppy version 5.0.12. The recovered data, which were obtained using NanoPlot (quality ≥ 9; length ≥ 500 bp; and head crop, 75 bp) (8), resulted in a mean read length of 74,804 bp and an *N*_50_ value of 132,120 bp. *De novo* assembly and polishing were performed using Flye ver. 2.8.3-b1695 (9), and genome completeness was confirmed using BUSCO version 4.1.2 (10). The genome sequence was annotated using DFAST ver. 1.2.6 (https://dfast.nig.ac.jp/) (11). The average nucleotide identity (ANIb) and digital DNA-DNA hybridization (dDDH) values were calculated using the JSpeciesWS online service version 3.9.8 (http://jspecies.ribohost.com/jspeciesws/#analyse) and the Genome-to-Genome Distance Calculator 3.0 (http://ggdc.dsmz.de/ggdc.php), with reported genome sequences of the type strains of the *L. buchneri* group. Default parameters were used for all software unless otherwise specified.

The complete MGR2-32 chromosome was 2,540,137 bp (44.0% GC content) and had a sequence depth of approximately 299×. In total, 1 contig region, 61 tRNAs, 15 rRNAs, and 1 CRISPR region were annotated. The pairwise ANIb and dDDH values between MGR2-32 and *L. buchneri* subsp. *silagei* SG162^T^ were 99.75% and 99.90%, respectively. The type strain and other strains of *L. buchneri* subsp. *silagei* have been isolated from silages in cold regions (1); however, strain MGR2-32 is unique in that it was isolated from tropical grass (5).

## Data Availability

The genome sequence and annotation data for strain MGR2-32 were deposited in DDBJ/GenBank under the BioProject number PRJDB13736, BioSample number SAMD00510914, DRA numbers DRA15835 and DRA15923; accession number AP028329; and SRA numbers DRX437897 (PromethION) and DRX435955 (Illumina NovaSeq).
